# Real-Time Multi-Modal Sensing and Feedback for Catheterization in Porcine Tissue

**DOI:** 10.3390/s21010273

**Published:** 2021-01-03

**Authors:** Christoff M. Heunis, Filip Šuligoj, Carlos Fambuena Santos, Sarthak Misra

**Affiliations:** 1 Surgical Robotics Laboratory, Department of Biomechanical Engineering, Faculty of Engineering Technology, University of Twente, 7500 AE Enschede, The Netherlands; f.suligoj@utwente.nl (F.Š.); carlosfambuena@gmail.com (C.F.S.); s.misra@utwente.nl (S.M.); 2 The Department of Biomedical Engineering, Faculty of Medical Sciences, University Medical Centre Groningen, 9713 GZ Groningen, The Netherlands

**Keywords:** image-guided surgery, medical robotics, multi-modal sensing, robotic registration

## Abstract

Objective: In this study, we introduce a multi-modal sensing and feedback framework aimed at assisting clinicians during endovascular surgeries and catheterization procedures. This framework utilizes state-of-the-art imaging and sensing sub-systems to produce a 3D visualization of an endovascular catheter and surrounding vasculature without the need for intra-operative X-rays. Methods: The catheterization experiments within this study are conducted inside a porcine limb undergoing motions. A hybrid position-force controller of a robotically-actuated ultrasound (US) transducer for uneven porcine tissue surfaces is introduced. The tissue, vasculature, and catheter are visualized by integrated real-time US images, 3D surface imaging, and Fiber Bragg Grating (FBG) sensors. Results: During externally-induced limb motions, the vasculature and catheter can be reliably reconstructed at mean accuracies of 1.9±0.3 mm and 0.82±0.21 mm, respectively. Conclusions: The conventional use of intra-operative X-ray imaging to visualize instruments and vasculature in the human body can be reduced by employing improved diagnostic technologies that do not operate via ionizing radiation or nephrotoxic contrast agents. Significance: The presented multi-modal framework enables the radiation-free and accurate reconstruction of significant tissues and instruments involved in catheterization procedures.

## 1. Introduction

X-ray fluoroscopy has been commonly used as a modality for visualizing endovascular catheters for arterial diagnosis and treatments owing to its high-speed display of complex vasculature. Specifically, it has led to the development of remote-controlled catheter navigation systems (RCCNS) such as Amigo remote catheter system (Catheter Precision Inc., Ledgewood, NJ, USA), Sensei robotic navigation system (Hansen Medical Inc., Mountain View, CA, USA), and Niobe (Stereotaxis Inc., St. Louis, MO, USA). These commercially-available systems have demonstrated not only that they can perform safely, but also that they improve the control and manipulability of catheters during endovascular interventions when compared to manually-controlled catheters [1,2]. Nonetheless, the control and positioning of catheters are still dependent on the expertise of the clinician and require long periods of specialized training time [3]. Moreover, despite the improvements brought by RCCNS to the field, these systems employ fluoroscopy images as their primary source of information, causing adverse effects to high-risk patients due to ionizing radiation exposure [4,5]. This results in limited periods of visibility of catheters inside the body, accompanied by additional challenges, such as those related to arterial inaccessibility and the lack of information about three-dimensional (3D) visual feedback [6].

Some studies have investigated the integration of intraoperative magnetic resonance imaging (iMRI) with RCNNS to reduce radiation exposure to patients and clinicians. Bell et al. developed a tendon-driven catheter that was visualized in real-time inside an MRI bore [7]. However, the catheter heated up close to the edge of the scanner bore and required a thick shaft diameter to be visible. This, in turn, increased the force necessary to control it, which reduced its positioning accuracy. Additional drawbacks of iMRI were noted when Liu et al. developed a framework that visualizes a robotic ablation catheter inside an MRI scanner [8]. They had difficulties with identifying the real-time shape of the catheter from MR images due to a low image acquisition rate. This limits automatizing catheter control strategies using iMRI, as substantiated by Alam et al.—a comprehensive study investigating multiple optical imaging techniques [9]. More notably, iMRI systems produce high magnetic fields that affect the electronics of electromagnetic actuators and sensors [10].

Several attempts have been made to bypass the use of iMRI systems by using either ultrasound (US) imaging [11,12] or Fiber Bragg Grating (FBG) sensors [13,14]. The latter has shown significant prospects in the real-time shape sensing of catheters [15,16,17]. However, these studies have not yet demonstrated real-time and simultaneous arterial and instrument 3D reconstruction. Alternatively, robotically-actuated US transducers can be employed for arterial reconstruction. Moreover, force/torque sensors can be integrated with transducers, since consistent contact force is required with the skin for visualizing blood vessels. Such strategies have shown potential for real-time automatic arterial characterization [18,19]. Mathiassen et al. further suggested the potential of robotic US path planning through force control for real-time 3D arterial reconstruction [20]. They proposed a hybrid-force sensing strategy on a stationary abdominal phantom, which has been adopted for autonomous US-guided tumor dissection [21]. However, this was not tested on heterogeneous tissue with uneven surfaces, which is a crucial challenge for a US-specific patient-oriented approach. This challenge was addressed by Graumann et al., who generated robotic US paths for covering a volume of interest selected in diagnostic images [22]. Nonetheless, they assumed that the US transducer is always positioned perpendicular to the tissue surface. A more desirable assumption would have been to position the transducer above the vessel of interest, as would be required to visualize catheters inside vasculature. Jiang et al. attempted to improve this framework by acquiring real-time US images through impedance control on the US transducer [23]. However, their method requires a full fan sweep motion of the transducer at each surface point to optimize the US transducer orientation alone. More importantly, none of these studies has incorporated representations of limb motions, for instance, Periodic Limb Movements (PLMs) [24]. Such movements affect the reconstruction accuracies of autonomous image acquisition frameworks that utilize robotic arms. PLMs that occur during interventions introduce real-time disturbances, which should be compensated for when arterial and instrument reconstructions are essential to the clinician.

The challenge of patient motions has been approached with non-invasive commercial systems. Tracking systems, such as Northern Digital Inc. (NDI) Polaris (Polaris Industries, Medina, MN, USA) [25,26] and the NDI Aurora electromagnetic (EM) tracking system [27] have been used to visualize surgical instruments in relation to anatomic structures [28]. However, considering that the operating room is a clustered environment and the capture volumes of these systems are small, the movement of surgical staff is confined. EM sensors have also been integrated into US transducers themselves, resulting in so-called Tomographic US (tUS) devices. One such device, the PIUR tUS Infinity (PIUR Imaging, Vienna, Austria), combines data from optical markers, EM sensors, and inertial sensors [29]. However, preliminary studies have reported drawbacks associated with freehand tUS scans, such as overlapping US slices and the loss of subsurface spatial information due to a change in applied force between the transducer and the skin [30]. Furthermore, in a clinical setting, metals can affect the accuracy of EM sensors [31].

In order to obtain information about catheters inside the human body, the need for integrating alternative imaging and sensing modalities remains paramount. Based on the aforementioned challenges of conventional approaches, further clinical needs have been identified: firstly, a safe solution for multi-modal sensing is required. Sub-systems can be integrated that eliminate excessive radiation resulting from fluoroscopy. Secondly, intra-operative visual feedback of both an endovascular catheter and vascular anatomy should be implemented. The scope for this requirement should be to assist clinicians during endovascular surgeries and catheterization procedures. In this study, we provide solutions to these needs without requiring intra-operative X-ray imaging. Specifically, we aim to generate an efficient US path to cover a surface while compensating for PLMs and adhering to prescribed contact forces and US transducer poses. This is accomplished by combining data from three imaging modalities (a US scanner, a 3D surface point-cloud camera, and motion capture cameras) with FBG sensing data of an endovascular catheter. This catheter is inserted into a porcine limb subjected to periodic motions. We show that FBG sensors remain a viable option for flexible catheters, due to their reliability in tracking without the need for line of sight. To the best of our knowledge, such a framework that covers the integration of the imaging and sensing technologies discussed herein does not exist yet. Hence, the contributions of this study are as follows:

1. Assembly and calibration of an endovascular catheter that is embedded with FBG sensors and infrared precision spheres, allowing for real-time feedback.
2. Introduction of a radiation-free intra-operative imaging framework for catheterizations.
3. Fully autonomous US acquisition directly performed by a robotic system with visual-servo (VS)-based compensation of externally-induced PLMs.
4. Real-time multi-modal sensing and 3D visualization of the vasculature, catheter, and surrounding surface tissue.

This paper is organized as follows: Section 2 describes the multi-modal system integration, the calibration of the imaging and sensing modalities, and the pre-operative US planning algorithms. In Section 3, the intra-operative planning phase is discussed. This phase includes the VS-based motion compensation and the real-time vasculature and catheter visualization. We then demonstrate the sensing and feedback framework in a clinically-relevant experiment, followed by the results and a discussion of these results in Section 4. Section 5 concludes this paper and provides directions for future work.

## 2. Pre-Operative Calibration and Planning

**Figure 1:**
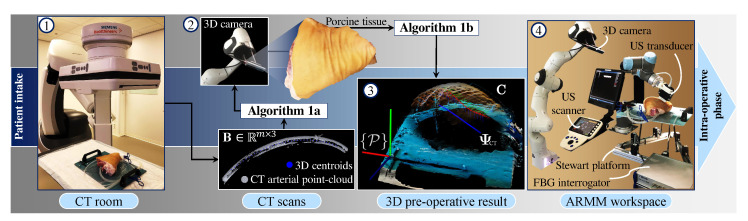
Pre-operative planning for the ultrasound (US) transducer. ① The porcine tissue is scanned pre-operatively with a computed tomography (CT) scanner. The resulting scanning data are converted to a point-cloud dataset ($B\in R^{m\times 3}$). 3D datapoints (*m*) are used as input to Algorithm 1, which calculates a set consisting of *n* arterial centroids ($\Psi_{\mathrm{CT}}\in R^{n\times 3}$) ② A second point-cloud ($C\in R^{r\times 3}$) is obtained from a 3D surface scan of the tissue and used as input to the Algorithm 2, the ultrasound pose planner. ③ The output is visualized as a set of poses consisting of projected surface via-points ($\Upsilon \in R^{n\times 3}$) and orientations ($\theta \in S^{n\times 3}$). ④ Finally, in the Advanced Robotics for Magnetic Manipulation (ARMM) workspace, a US sweep can be performed across the tissue, followed by multi-modal feedback of the 3D artery, tissue surface, and the Fiber Bragg Grating (FBG)-embedded catheter in the intra-operative phase.

This section provides an overview of the sub-systems used in this study—specifically with the intention to guide clinicians to utilize FBG-embedded catheters in a real-time environment. This is followed by a description of the multi-modal calibration process, US planning, and reconstruction algorithms. The workflow of the pre-operative phase is illustrated in Figure 1.

### 2.1. Imaging and Sensing Modalities

This study is performed in the Advanced Robotics for a Magnetic Manipulation (ARMM) system [32]. The framework discussed in this study is implemented on the ARMM workstation (Precision Tower 7910, Dell Technologies, Austin, TX, USA) running Ubuntu 18.04 (Kernel 4.4.236) and an RT-PREEMPT patch for real-time visualizations. A Point-Cloud Library (PCL) package (Willow Garage, Menlo Park, CA, USA) is used for the 3D rendering of point-clouds, while OpenCV (Open Source Computer Vision Library, v.3.4.2) is used for image processing. The modalities used in this study are as follows: A 6 degree-of-freedom (DoF) serial-link robot (Model UR5, Universal Robots, Odense, Denmark) is used to maneuver a linear US L14-5 transducer connected to a SonixTouch Q+ US scanner (BK Medical, Quickborn, Germany). Furthermore, a passive robot (Panda, FRANKA EMIKA GmbH, Munich, Germany) is used to hold and adjust the pose of a 3D depth camera (Intel Realsense SR305, Santa Clara, CA, USA). The depth camera reconstructs a 3D point-cloud structure of any surface at a rate of 60 Hz, and the transducer captures and streams 2D US images at a rate of 30 Hz. Motions of the catheter and tissue inside the ARMM workspace are recorded by an Optitrack Flex13 (NaturalPoint Inc., Corvallis, OR, USA) infrared precision sphere-based motion capture system. The endovascular catheter shaft is embedded with an optic fiber containing FBG sensors and connected to an FBG-Scan 804D interrogator (FBGS Technologies GmbH, Jena, Germany). These sensors provide 3D catheter reconstruction in a real-time environment.

In addition to employing these imaging and sensing modalities, this framework is demonstrated with porcine tissue, thus ensuring a near-realistic representation of a human limb. PLMs that mimic involuntary limb movement are simulated by a 6-DoF Stewart platform. This movement is compensated during US image acquisition by controlling the US transducer with a hybrid position-force controller designed for uneven body tissue surfaces. The implemented methods within this framework encapsulate the pre-operative planning of the arterial model of the porcine tissue, followed by the real-time intra-operative visualization during an endovascular catheterization procedure.

### 2.2. FBG-Embedded Catheter Assembly

**Figure 2:**
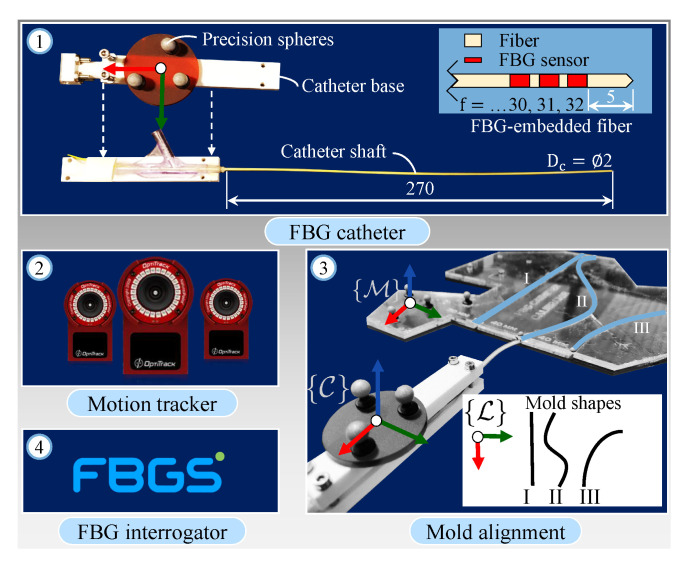
Assembly and calibration protocol for the Fiber Bragg Grating (FBG)-embedded catheter: ① 32 FBG sensors are inside a multicore fiber that is inserted in, and fixed to, the catheter shaft. The catheter base has a known offset with respect to the first sensor (*f* = 1). ② A rigid body precision sphere tool on the catheter base is tracked by eight infrared cameras that surround the Advanced Robotics for Magnetic Manipulation (ARMM) workspace. ③ A mold containing three channels with known coordinates in local frame ({L}) is used to calibrate the shape of the catheter to its base, ({C}) recorded in the global reference frame ({G}). Frame {M} is recorded via precision-spheres to obtain the pose of the mold. The straight channel (I) acts as a reference with a zero strain shift of the FBG fiber. The second channel (II) is used to calculate the transformation ($H_{L}^{M}$). The final channel (III) is used to validate the global transformation between the FBG coordinates streamed by ④, the interrogator and both the local channel shape ($H_{M}^{G}$), and catheter base frame ($H_{C}^{G}$). This information is combined to display the catheter shape in real-time. All dimensions are in mm.

The catheter assembly (Figure 2, number ①) consists of an endovascular catheter ($D_{c}=2$ mm diameter, 270 mm length) made from Polyethylene terephthalate (PET). This material has high stiffness and is chosen due to the curvature constraint imposed by the FBG fiber, which has a maximum bending radius of 50 mm. The multicore fiber (f=32 FBG sensors, 125 μm cladding diameter) is embedded into the central lumen of the catheter shaft. Precision spheres with 3 M 7610 reflective tape (Engineering Systems Technologies GmbH, Kaiserslautern, Germany) are then attached to the catheter base. This is triangulated by the motion capture cameras in the calibration phase using a Software Development Kit (SDK) from Motive NatNet (NaturalPoint Inc., Corvallis, OR, USA) and represents the catheter reference frame ({C}) inside the ARMM workspace.

### 2.3. Calibration of the Imaging and Sensing Modalities

The motion tracker frame of origin is chosen as the global reference frame ({G}) (Figure 2, number ②). All homogeneous transformations between the modalities and the motion tracker are derived to map their local reference frames to {G}. These transformations are calculated as

(1) $$\begin{array}{c} H_{b}^{a}=\left[\begin{array}{cc} R_{b}^{a} & p_{b}^{a}\\ 0_{1\times 3} & 1 \end{array}\right]\in \mathrm{SE}\left(3\right), \end{array}$$

where $R_{b}^{a}\in \mathrm{SO}\left(3\right)$ is a rotation matrix describing the relative orientation of a frame ({b}) with respect to another frame ({a}) and $p_{b}^{a}\in R^{3}$ is the translation vector from a point in frame *a* to frame *b*. In this study, we utilize the precision spheres to calibrate the FBG sensors with the catheter base, in addition to tracking it with the motion capture system.

**Figure 3:**
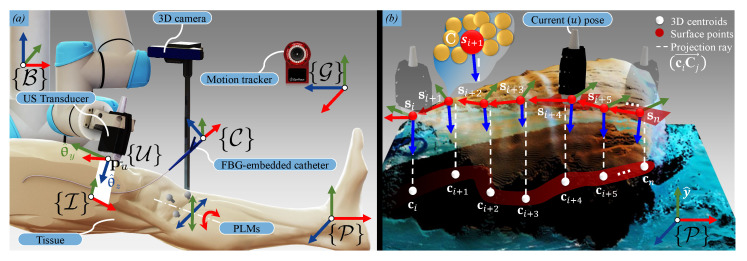
Intra-operative ultrasound (US) transducer pose planning. (**a**) In this study, we integrate three imaging modalities: a 3D depth camera, a US transducer, and a motion tracking system. Additionally, data from the Fiber Bragg Grating (FBG) sensors are fused with imaging data to provide real-time shape information of the endovascular catheter. This information is combined with the reconstructed environment from the pre-operative planning to visualize the catheter inside the arterial volume, which is subjected to Periodic Limb Movements (PLMs). All reference frames are indicated in brackets ({}). (**b**) Surface points ($s_{i}$) are obtained by vertically projecting the arterial centroids ($c_{i}$) obtained from Algorithm 1, and finding the closest point on the surface point-cloud dataset ($C_{j}$). A trajectory for the US transducer is then calculated along the tissue surface. A proportional-integral (PI) controller positions the transducer, while its orientation is controlled by a P-controller. Together, these controllers realize the pose of the transducer (obtained from Algorithm 2) at a 3D surface target point.

In order to calibrate the catheter, we first determine the configuration of the catheter shape in the global reference frame ({G}). This is done through a mold alignment process. In this process, the catheter is inserted into three different channels with known curvature (Figure 2, number ③. The mold reference frame ({M}) is registered in the global coordinate frame ({G}) using Motive NatNet. Following this registration, the catheter is inserted into three mold channels with local design frame ({L}), while its shape and base frame pose are simultaneously recorded by the FBG sensors [33] and motion capture system, respectively. The catheter shape is reconstructed in 3D Euclidean space as a point-cloud dataset ($\Phi_{F}\in R^{3}$). Utilizing (1), the catheter shape in the global frame ($\Phi_{G}\in R^{3}$) is calculated by

(2) $$\begin{array}{c} \Phi_{G}=H_{C}^{G}H_{F}^{C}\Phi_{F}, \end{array}$$

where $H_{F}^{C}$ is calculating during the calibration procedure. Next, we register the robot base frame ({B}) and end-effector (i.e., the US transducer) frame ({U}), to the global frame through accurate full pose measurements (position and orientation) of the end-effector in Cartesian space. These measurements result in obtaining $H_{U}^{G}$ and $H_{B}^{G}$. The final calibration is required to localize the polar coordinate US data (pixels) to Cartesian coordinates using a custom-designed calibrator object. For this calibration, the US image plane is mapped to obtain $H_{I}^{U}$, where {I} is the image reference frame. All frames are displayed in Figure 3a. Details regarding these calibration procedures can be referred to in the Supplementary Materials. Following these calibrations, the imaging and sensing modalities are ready to be used in the pre-operative planning phase.

**Algorithm 1** 3D centroid generation inside an arterial volume

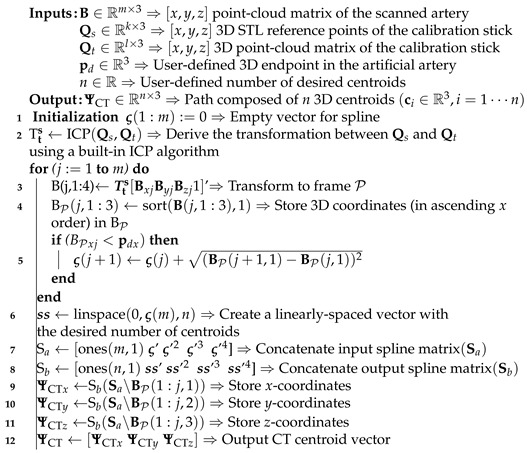



**Algorithm 2** Robot-mounted US transducer pose generation

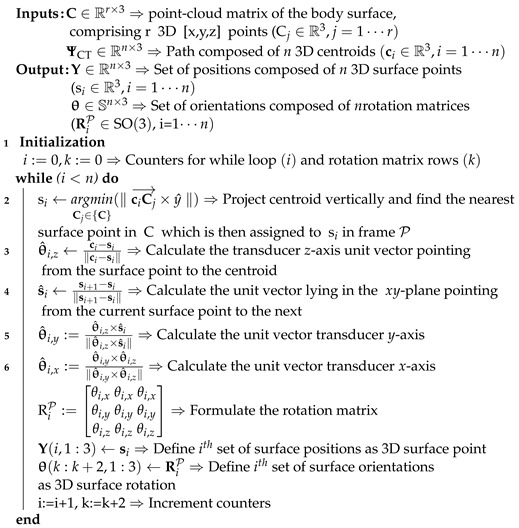



### 2.4. Pre-Operative Planning

Precision spheres are rigidly attached to a 3D-printed calibration stick that represents the reference frame ({P}) of the porcine tissue for tracking purposes. We use an Artis Pheno robotic C-arm cone-beam scanner (Siemens Healthcare GmbH, Forchheim, Germany) to scan the porcine tissue. Open-source software (InVesalius, Renato Archer Information Technology Center, Campinas, Brazil) is then utilized to eliminate soft tissues and strip the bone structure, keeping the vasculature and the calibration stick as two separate mesh objects. These anatomical details are converted to point-cloud data structures and used as input to both the arterial centroid generator (Algorithm 1) and the US pose planner (Algorithm 2). The centroid generator is implemented to obtain data points that correspond to the CT slices along the global longitudinal axis (i.e., along the length of the artificial artery). This algorithm processes three sets of data points: Imported 3D points ($Q_{s}\in R^{k\times 3}$) of the calibration stick Standard Triangle Language (STL) data, the calibration stick mesh ($Q_{t}\in R^{l\times 3}$), and the point-cloud mesh ($B\in R^{m\times 3}$) of the vasculature, where k,l, and *m* are the number of data points. Points within $Q_{s}$ and $Q_{t}$ are processed only once to derive the transformation matrix ($H_{t}^{s}$) between the CT slices and the tissue reference frame. This is done through an iterative closest point (ICP) algorithm to compute a matching that minimizes the root mean squared distance between these two point-sets [34]. Next, a fourth-order polynomial representation ($P\left(v\right):R\to R^{3}$) of the artificial arterial centerline is derived and discretized to obtain a set of *n* centroid positions within the arterial volume. These centroids are individually defined as $c_{i}\in R^{3}$ (i∈1,...,n) and form part of a subset ($\Psi_{\mathrm{CT}}\in R^{n\times 3}$) consisting of *n* user-defined CT points.

Subsequently, the US pose planner Algorithm 2) is executed. The input to this algorithm is the point-cloud dataset ($C\in R^{r\times 3}$) of the tissue surface. To obtain this point-cloud, the 3D depth camera is positioned above the tissue. A 3D RGB-D image is captured and rendered as an xyz-point-cloud structure using the PCL interface. Once both the arterial centroids and the surface point-cloud are transformed into the same coordinate system ({P}), Algorithm 2 uses the CT-derived subset ($\Psi_{\mathrm{CT}}$) and C as an input to calculate a trajectory for the US transducer on the surface of the tissue. Recalling that the US transducer should be positioned above the centroid ($c_{i}$), these centroids are first projected upwards in the porcine tissue *y*-axis (Figure 3b). The nearest point ($C_{j}\in \left\{C\right\}$) found on the surface to the ray ($\vec{c_{i}C_{j}}$) then becomes a surface path point ($s_{i}\in R^{3}$). For each surface point, a rotation matrix ($R_{i}^{P}\in \mathrm{SO}\left(3\right)$) is also calculated, defining the transducer *z*-axis as the vector pointing from the surface point to the centroid. An additional vector lying on the xy-plane is derived from two consecutive surface points ($s_{i}$ and $s_{i+1}$). The final output of the algorithm is the US transducer pose defined for each of the *n* corresponding arterial centroids ($\Psi_{\mathrm{CT}}$), comprising a set of positions ($\Upsilon \in R^{n\times 3}$) and orientations ($\theta \in S^{n\times 3}$) in the tissue reference frame. With this data in place, the porcine limb is positioned in the ARMM workspace.

## 3. Multi-Modal Sensing and Feedback

This section describes the second half of our framework—a real-time data acquisition protocol and multi-modal feedback via a visualization interface. We begin by describing the position-force hybrid controller and our VS approach. This is then followed by the image processing of US images and reconstruction of the catheter shape for real-time visualization.

### 3.1. Visual-Servo-Based Motion Compensation

A custom-built 6-DoF Stewart platform is used to introduce PLMs to the tissue. This platform functions independently from the VS-controller and is controlled by six servo motors (MX-64AR Dynamixel, Robotis, Korea). Reflexive PLMs in patients have been reported to occur approximately every 30 s, though they have no predictive validity [35]. Hence, we assume that the disturbance is periodic. The model that describes the periodic motion of the tissue is given by a two-term Fourier series (see Supplementary Materials). Second, we only introduce translational motions in the tissue zy-plane, and rotation about the *x*-axis, via reference signals that describe PLMs that are motion-constrained along the length of a leg. These signals are recorded via precision spheres by the motion-capture system at a rate of 120 Hz in the global reference frame ({G}).

The transducer poses acquired from Algorithm 2 are used to control the robotically-actuated US transducer at each surface point on the tissue surface with the assumption that the tissue is stationary. Thus, when motions occur, an updated pose should be calculated and used as input to the robot. The current (*u*) robotic end-effector pose at any specific instant is expressed in terms of the transducer position ($p_{u}\in R^{3}$) and angle-axis orientation ($\theta_{u}\in S^{3}$) in the robot base frame ({B}). Therefore, according to (1), we first calculate the transformed pose ($H_{i}^{P}=\left[R_{i}^{P},p_{i}^{P}\right]$). Specifically,

(3) $$p_{i}^{P}=\left[\begin{array}{c} s_{ix}-o_{z}\\ s_{iy}+o_{y}\\ s_{iz}+o_{x} \end{array}\right],$$

where $o_{x},o_{y}$, and $o_{z}$ represent the real-time displacements of the precision spheres (obtained from the Motive NatNet data stream and transformed to the tissue reference frame). Second, the updated target surface position ($\Delta s_{i}$) is obtained from the translation component of the transformation matrix ($H_{i}^{B}={H_{B}^{G}}^{-1}H_{P}^{G}H_{i}^{P}$). Here, $H_{B}^{G}$ and $H_{P}^{G}$ represent the homogeneous transformation matrices calculated according to (1), and $R_{i}^{P}$ from Algorithm 2, line 7 is updated by the rotation of the tissue in space. Concurrently, the position-orientation controller is activated for the US transducer. Let us consider the transducer leaving a current (*u*) pose to reach a target (*i*) pose. Then, given the current position ($p_{u}$) of the transducer, the position error ($E_{p}\in R^{3}$) and orientation error ($E_{\theta }\in R^{3}$) are calculated. These errors are minimized by a task-space velocity controller, outlined in Appendix A, which allows the transducer to reach a target.

When the transducer approaches a compliant environment, i.e., the surface of the porcine tissue, the force-based explicit force controller is active. Force-feedback is provided by a three-axis force sensor (K3D40, Mesysteme AG, Henningsdorf, Germany) connected between the transducer and the robot tool flange. Forces are only measured until conditions describing a positioning threshold, ($|E_{p}|<d_{p}\in R$), orientation threshold, ($E_{\theta y},E_{\theta z}<d_{\theta }\in R$) and a force error ($E_{f}\in R^{3}$) have been met. The force error is calculated as the difference between the normal force of the tissue against the transducer surface and the desired force ($d_{f}\in R$). The desired force is chosen such that sudden surface deflection is avoided, and constant contact is maintained. Satisfying all feedback conditions, such as the contact force ($d_{f}$), positioning threshold ($d_{p}$), and orientation thresholds ($d_{\theta }$), implies that the transducer is positioned both on the surface point ($s_{i}$) and above an arterial centroid ($c_{i}$) and, hence, a US image is acquired.

### 3.2. Ultrasound-Based Arterial Reconstruction

**Figure 4:**
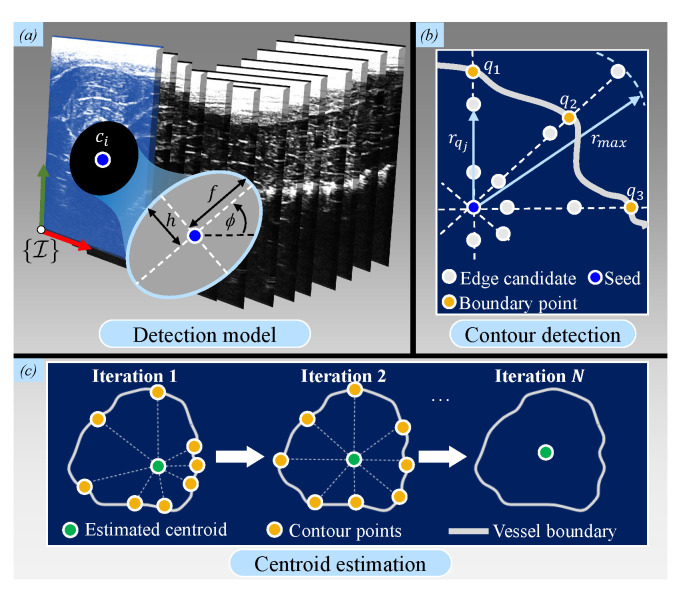
Ultrasound (US) image arterial reconstruction scheme. (**a**) For each US image slice, the arterial contours are extracted and reconstructed centroids ($c_{i}$) are estimated. Knowledge of the ellipse parameters (*f*, *h*, and ϕ) are required and included in an extended Kalman filter; (**b**) equi-spaced edge candidates along a radial line with maximum predefined length ($r_{max}$). For each candidate (${}_{j}$) in line (*q*), the distance to the center along this line is defined as $r_{q_{j}},j\in N\;\left(j=1,\ldots ,N\right)$. (**c**) Each centroid of the US slices is estimated at each iteration (*q*), repeated iteratively, until the calculated center converges to the weighted center.

Brightness (B)-mode images are acquired at each surface point and converted to 2D US images which are then processed to obtain arterial shape information. This information consists of reconstructed arterial contour points (set $\Lambda_{\mathrm{US}}\in R^{N\times 3}$) and centroids (subset ($\Psi_{\mathrm{US}}\in R^{n\times 3}$)). The boundary of the contour is estimated by a probabilistic edge-detection filter [36]. This information is combined with the predictions of an ellipse model, which are assumed to be suitable for semi-circular arteries. Generally, for each US slice, we describe an arterial contour using the nonlinear dynamic system:

(4) $$\left\{\begin{array}{c} x_{q+1}=x_{q}+\xi_{q},\\ r_{q}=D\left(x_{q}\right)+\eta_{q}, \end{array}\right.$$

where $r_{q}\in R$ is the output radius length and q∈N represents each of *N* indices around the arterial centroid for which the states of the system ($x_{q}\in R^{3\times 1}$) are estimated. These states are the lengths of the semi-major axis ($f_{q}\in R$) and semi-minor axis ($h_{q}\in R$) axes, as well as the tilt angle ($\phi_{q}\in R$) of the ellipse (Figure 4a). Next, as shown in [36], we assume white, zero-mean Gaussian process for the sequence ($\xi_{q}$) and measurement noise ($\mu_{q}$) with known covariances which are constant throughout the entire contour. The tuning of the covariance values is done empirically until the estimated contours adapt well to the actual boundaries of the artery. The calculations of the model ($D(x_{q})$) and covariances are provided in Appendix B.

When each contour point has been defined, the arterial centroids ($\Psi_{\mathrm{CT}}$) in the subsequent iterations are calculated by means of a Star Algorithm [37]. This algorithm calculates the center of mass of all contour points (Figure 4c). After the final iteration, each centroid is stored in the subset ($\Psi_{\mathrm{CT}}$). All centroids are then compared to the previously-obtained ground truth centroids ($\Psi_{\mathrm{CT}}$) for further validation.

### 3.3. Catheter Shape Reconstruction

The arterial reconstruction is then followed by localizing the polar coordinate US data to Cartesian coordinates for real-time feedback. First, homogeneous representations of the set of *N* US contour positions ($\Lambda_{\mathrm{US}}\in R^{N\times 4}$) are transformed to the global reference frame ({G}) by concatenating matrices described by (1) as follows:

(5) $$H_{\mathrm{US}}^{G}=H_{B}^{G}H_{U}^{B}H_{I}^{U}\Lambda_{\mathrm{US}},$$

which also applies to the arterial centroids ($\Psi_{\mathrm{US}}$) to obtain $\Psi_{G}$. These points are visualized in 3D space to provide feedback on the geometry of the artery. The final step is to visualize the catheter shape ($\Phi_{G}$) obtained from (2) inside this artery. The catheter shape information is again obtained using the FBG sensor data. By transforming this data to the reference frame of the catheter base (as discussed in Figure 2), a real-time representation of the catheter shaft is obtained in the global frame ({G}). Furthermore, contact points between the catheter shaft and the inner arterial wall (spline-fitted contour points) are estimated. Since we do not account for soft-tissue deflections in this study, we determine these contact points in a non-deformable mock-up phantom of the porcine artery. After implementing the discussed framework on this mock-up, these contacts are determined by estimating the Euclidean distances between the mock-up boundary and the FBG sensor positions in 3D. Once these distances are below a certain threshold, contacts are visualized.

## 4. Experimental Results

This section describes the experimental setup used to validate our multi-modal sensing and feedback framework. The accuracy of US reconstruction is quantified using the CT-generated point-cloud data as a ground truth. This is followed by the estimation of contact points between the catheter shaft and the inner arterial wall, which is validated within the mock-up phantom of an artificial porcine artery. Finally, we validate the overall reconstruction accuracy of an artificial artery that is inside the porcine tissue.

### 4.1. Procedure

Catheter insertion is performed on a freshly excised porcine hind limb obtained from a local slaughterhouse (Figure 1, number ④. We prepare the limb by making incisions below the knee and above the hip. In order to establish artificial blood flow, a flexible silicone tube (10 mm inner diameter, 1 mm thickness) with similar dimensional characteristics of a femoral artery is inserted into the limb [38]. The limb is scanned with the CT scanner, and the DICOM output data file is imported into the ARMM Graphical User Interface (GUI) [32]. This GUI converts anatomical details to point-cloud data structures which are automatically processed by Algorithms 1 and 2 for n=16 setpoints. Next, the pre-operative planning phase (Section 2.4) is validated by performing a US sweep on the stationary (ST) limb. The US focus depth is set to 20–40 mm, with a maximum depth of 90 mm and 10 Mhz resolution. The position-orientation and force-control parameters for the US transducer are shown in Table 1. For the 10 mm diameter tube representing the artery, we choose boundary detection indices as N=30.

Subsequently, multi-modal feedback and visualization (Section 3) are demonstrated during PLMs, which are produced by the 6-DoF Stewart platform and compensated for by the VS controller. The desired US transducer poses and trajectories are calculated for a segment of the artificial artery (length 125 mm), followed by arterial visualization. Finally, insertion is done with the FBG-embedded catheter, which is visualized inside the artificial artery on the PCL interface in conjunction with the tissue surface. Please refer to the accompanying video (https://www.dropbox.com/s/643q13ixv52oavn/ARMM_Multimodal_Systems.mp4?dl=0) to view this procedure.

### 4.2. Results

**Figure 5:**
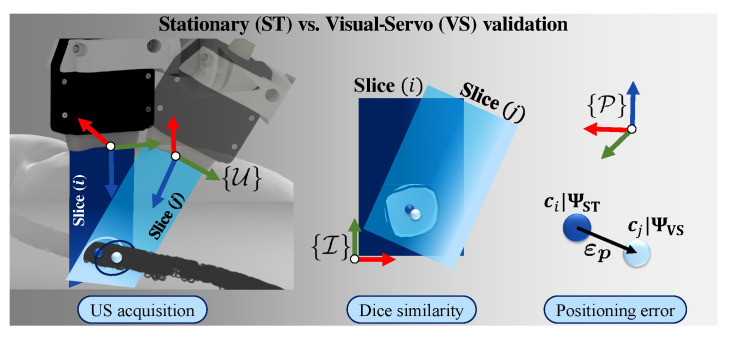
Calculating the reconstruction accuracies: The US images are paired as slices pertaining to each via-point on the skin surface in the transducer frame ({U}). Dice similarities are calculated between stationary (ST) US slices and dynamic slices captured during the visual-servo (VS) trial, both in the image reference frame ({I}). Finally, the 3D Euclidean errors ($\varepsilon_{p}$) are calculated, using the centroids ($c_{*}$) of each slice (∗=i,j) as reference point in frame ({P}).

**Figure 6:**
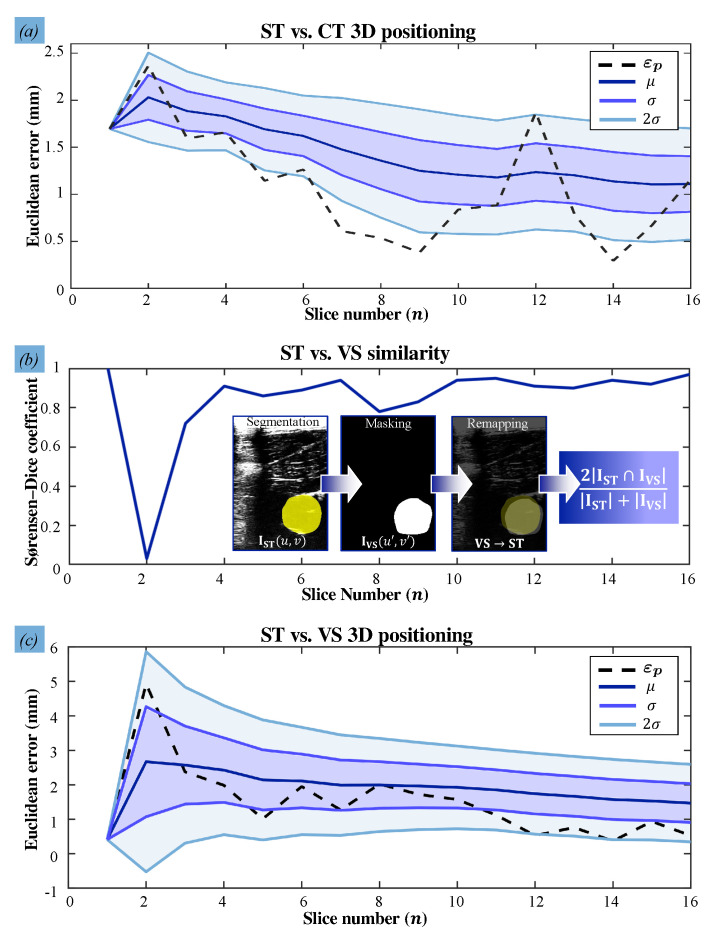
3D Euclidean errors ($\varepsilon_{p}$) and reconstruction results: (**a**) stationary US image centroids are compared to the ground truth centroids obtained during the Computed Tomography (CT) scan. The mean positioning error (μ), single (σ), and double (2σ) measurement of variations are indicated for each slice (*n*). (**b**) The Dice similarity results are shown, comparing the binary masks of the segmented US image ($I_{*}$) pairs for the two trials (${}_{*}=\mathrm{ST},\mathrm{VS}$). Each mask is mapped in image coordinates (u,v). (**c**) Finally, the 3D Euclidean errors are reported, comparing the US transducer positioning accuracy of the ST and VS acquisition trials.

The accuracies at which the US images and the catheter shape are reconstructed in the stationary and VS experiments are calculated (Figure 5). In order to first validate the accuracy of the US reconstruction, we calculate the 3D Euclidean errors of the reconstructed arterial centroids and quantify the mean and mean absolute deviations of these errors. During the stationary trial, the arterial centroids ($\Psi_{\mathrm{US}}$) are compared with the ground truth data obtained from the CT slices ($\Psi_{\mathrm{CT}}$) resulting in a mean spatial error of 1.1±0.5 mm. With PLM-induced disturbances, the arterial centroids ($\Psi_{\mathrm{VS}}$) deviate from the actual centroids ($\Psi_{\mathrm{US}}$) at a mean positioning error of 1.9±0.3 mm. These results show the desired correspondence of the reconstructed US artery with the ground truth (Figure 6).

**Figure 7:**
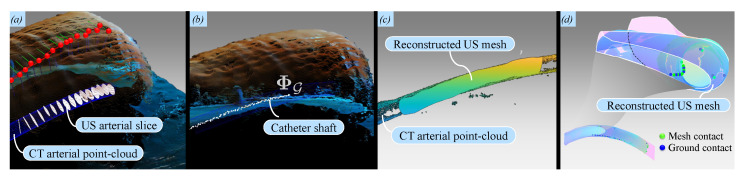
Multi-modal visualization results: (**a**) The point-cloud library (PCL) interface is used to visualize the porcine tissue surface, red projected surface points, the ultrasound (US) transducer orientation frames, and the reconstructed US slices (white); (**b**) visualization of the Fiber Bragg Grating (FBG)-embedded catheter ($\Phi_{G}$) inside the artificial artery; (**c**) the reconstructed US artery is reproduced as a surface mesh comprising centroids and boundaries. Visual inspection of the reconstruction of the artificial artery yields commendatory results when compared to the Computed Tomography (CT) point-cloud; (**d**) Potential contact points between the catheter shaft and the artificial artery inner wall are estimated using an approximative method to threshold their Euclidean distances. For the current catheter (with a radius of 1 mm), this threshold is chosen as 1.25 mm. Please refer to the accompanying video (https://www.dropbox.com/s/643q13ixv52oavn/ARMM_Multimodal_Systems.mp4?dl=0) to view this visualization.

Next, we validate the reproducibility of US images and the consistency of the transducer orientations. The reproducibility concerns the amount of overlap between two US images of the same setpoint during and without motions. This overlap is obtained by deriving the Sørensen–Dice coefficient of the corresponding 2D masks of 16 reconstructed US slices. The mean similarity between US slices is 0.84. Specifically, in Figure 6c, we observe that the controller reliably compensates for disturbances only from the second via-point, shifting from 1.00 (n=1) to 0.02 (n=2). We attribute this to the gradual, low change in tissue-transducer contact force that occurs at the second via-point. This is in contrast to the high, sudden transition between position and force control at the first via-point. This outlier can be improved by manually positioning the transducer close to the first surface point before commencing the US sweep. Overall, the controller adapts quickly to sudden changes in the phantom pose, as robot joint velocities take an average of 6 ms to be calculated and prescribed. Finally, catheter shape information is retrieved from FBG sensors embedded in the catheter shaft and successfully visualized (Figure 7). The shape is represented as a point-cloud that encapsulates the measured FBG sensor positions in 3D. The mean Euclidean error between the reconstructed coordinates of the FBG sensors and those measured by the FBGS interrogator is 0.82 mm, with a maximum error of 1.52 mm. Finally, we have demonstrated that FBG sensors also aid with the identification of potential contact points inside the artery.

### 4.3. Error Analysis

Sources of the cumulative localization error are divided into those concerning arterial and catheter reconstructions, respectively. Reconstructing the arteries result from relative motion matrices presented (5), during which errors can accumulate. These sources include the in-image localization errors and robot positioning error (0.3±0.1 mm) of which 90% are attributed to robot geometric errors [39]. Catheter reconstruction errors originate from the FBG sensor reflectivity and the uncertainties from precision sphere triangulation. A higher reflectivity of the sensors would result in more accurate detection of the Bragg wavelength and, hence, reduced error [33]. Moreover, triangulation errors occur due to the calibration of the motion tracking system, which has been measured for the ARMM system as shown in [40]. The triangulation error is 0.56±0.08 mm, which is the mean Euclidean distance between the coordinates in the global reference frame ({G}) and their reprojections. Quantification of these calibration errors is explained in more detail in the Supplementary Materials (Table S1).

Due to the nature of soft tissue, reconstruction errors are expected to be inconsistent and dependent on the precision limitations of the sub-systems. Such errors are important to consider for measurement accuracy of arterial geometry, and guiding catheters using sub-surface imaging. Apart from the reported mean positioning errors between estimated and actual spatial coordinates, the reconstruction errors are fitted to two realistic scenarios. In the first scenario (**S.A**), the dichotomous independent variable is chosen as a threshold of 1.12 mm, defining whether the error is clinically-acceptable or not [41]. In the second scenario (**S.B**), this variable is defined by the length of a US sweep, which we believe influences the accuracy due to robotic drift. We arbitrarily choose the half the distance traveled along the segment of the porcine artery (125 mm length) as the threshold.

Our dependent variable is constructed by three categories regarding arterial reconstruction errors that would influence catheter tip placement for both scenarios. These categories are based on bounds $\left(\varepsilon_{p}\in \left(D_{c}/2,D_{c}\right)\right)$ of the catheter diameter, with a low error of (≤$D_{c}/2$), a medium error within these bounds, and a high error (≥$D_{c}$). Based on these variables, uncertainties are calculated to understand the reliability of the results. We perform a Proportional Reduction in Error (PRE) analysis in SPSS Statistics (IBM, New York, NY, USA) for each scenario (Table 2). This analysis delivers uncertainty coefficients of 0.618 and 0.261, respectively. For scenario **S.A**, this implies that choosing a clinically-accepted threshold reconstruction reduces the probability of a prediction error by 61.8%. In addition, having knowledge of the artery length in scenario **S.B** improves the probability of predicting the correct error by 26.1%. Finally, the US reproducibility validation metric of 0.84 is quantified for stationary measurements, resulting in a mean Dice coefficient of 0.96. According to [42], any coefficients higher than 0.7 are regarded as an excellent agreement.

## 5. Conclusions and Future Work

In this study, we present a clinically-relevant 3D visualization framework for an autonomous robotic system that integrates multiple imaging sub-systems with FBG sensing technology. By means of multi-modal sensing, the robotic system provides feedback of an FBG-embedded catheter and 3D tissue surfaces in real-time while compensating for uncertainties such as PLMs. The controlled synchronization of a serial-link robot with a moving limb is achieved. The stabilized images of the limb tissue surface, vasculature, and catheter are presented to the operator, allowing for catheterization in a virtually-motionless limb. We experimentally evaluate the reconstruction accuracy of the system in motionless and non-static scenarios, resulting in mean positioning errors of 1.9±0.3 mm and 0.82±0.21 mm for the reconstructed arteries and catheter, respectively.

### 5.1. Current Limitations and Clinical Feasibility

While the framework presented in this study is reliable in terms of its reconstruction accuracies, several findings have been identified that have important implications for developing a clinic-ready system. Firstly, whether the reported errors are considered acceptable depends on the application for vascular surgeries. The acoustic lens of the L14-5 transducer allows for the visualization of all arterial diameters, since the maximum reported diameter (24 mm) is that of the aorta [43]. In contrast to neurosurgeries, where cerebral vein diameters are much smaller, the consensus among vascular surgeons is that submillimeter reconstruction accuracy is not necessarily required [44]. Nonetheless, the maximum tracking accuracies of commercial RCCNS systems are ≥4 mm [45], making the current framework still comparable with the state-of-the-art.

Secondly, the integration of numerous sub-systems may be tedious and difficult to implement during point-of-care diagnosis. The ARMM GUI can alleviate this difficulty since it allows clinicians to experience surgical practice by delivering visual and sensing information. Furthermore, some of the presented sub-systems can be reduced or replaced. A reliable alternative for the US transducer robot arm would be a redundant 7-DoF robot such as the Panda (Franka Emika GmbH, Munich, Germany) or the LBR IIWA robot (Kuka, Augsburg, Germany). These robots contain integrated torque sensors which eliminate the need for explicit force control, as an impedance controller can then instead be utilized in conjunction with the 3D camera. Furthermore, the 3D camera can be employed for both the purpose of streaming topographical landmarks and their poses, thereby potentially eliminating the need for the motion-capture system.

Thirdly, this framework is regarded as safe and effective from a risk perspective. Catheters and surrounding arteries can be visualized to a clinician with low error, and indicate a strong potential of this framework towards virtual stabilization in a surgical environment. However, there may still be cases in which intra-operative X-rays are required to visualize vascular structures at the target site or observe clinical procedures that are more intricate. Notwithstanding, the use of US images can bypass this requirement [46]. Since US images and motion data are captured in real-time, this framework could help clinicians visualize the vascular target and particular tools, such as stents, ablation tips, and angioplasty balloons. This framework can aid with this visualization, given that this target is known a priori. It is recommended that visualization should remain without intra-operative X-ray imaging, unless mechanical complications arise.

Finally, the maneuverability of current FBG-embedded catheter is imposed by a design constraint: the bending radius of the FBG fiber. For vascular catheterizations—especially those relating to cardiac disease—arteries may be more tortuous than those presented in this study. Fortunately, other FBG fibers exist which can be integrated with catheter shafts with more resilience. Fibers with higher core aperture values and polyimide protective coatings have been reported with bending radii of 2.6 mm [47]. Such fibers can replace the one presented in Figure 2, in addition to choosing catheters based on the distance of vascular lesions, the tortuousness of the route, and the diameter of the vasculature.

### 5.2. Future Work

In future studies, this framework will be utilized to allow for improved control over the inner arterial positioning of catheters within the ARMM system. Catheters will be guided in this system by means of magnetic actuation [32], followed by the demonstration of a specific function (e.g., atrial fibrillation, angioplasty, or atherectomy techniques). The contact-point formulation can be expanded in an attempt to combat undesirable catheter-tissue friction during insertion. Furthermore, this framework would serve as a baseline upon which more complicated diagnostic capabilities can be built, for example, segmenting different layers of body tissue by exclusively processing US images and 3D depth camera data. This would require improvements that enable the 3D reconstruction of the entire US volume, as opposed to separate slices in that volume. Finally, different diameters of vasculature should be tested, since this framework could then be applicable during the treatment of brain and spinal cord aneurysms, which consist of much smaller vascular networks than the size of those investigated in this study. It is recommended that this framework should be tested by clinicians who conventionally utilize X-ray fluoroscopy, and compare the results to report on its feasibility. Furthermore, the reconstruction errors should be evaluated through practice-based statistical analysis as explained by [48], which would require a large number of observations through US sweeps over larger distances than those demonstrated in this study. 

## Figures and Tables

**Table 1:** The position, orientation, and force control parameters for the robotically-actuated ultrasound (US) transducer. Each parameter can be referred to in Appendix A and Appendix B.

Control Parameter		Symbol	Value
**Position**	Positioning setpoint	$d_{p}$	0.5 mm
	Velocity threshold	μ	25 mm
	Integral time constant	τ	20
	Maximum transducer linear velocity	$V_{d}$	50 mm\/s
**Orientation**	Orientation setpoint	$d_{\theta }$	0.01 rad
	Orientation threshold	γ	0.1 rad
	Maximum transducer angular velocity	$\omega_{d}$	0.1 rad/s
**Force**	Contact force setpoint	$d_{f}$	0.8 N
	Proportional gain	$K_{p}$	0.5
	Integral gain	$K_{i}$	0.7

**Table 2:** Directional measures of association between two scenarios (**S.A** and **S.B**) and the reconstruction 3D errors. Uncertainty coefficients of 0.618 and 0.261 are estimated and considered to be statistically significant (p<0.01 for **S.A** and p<0.05 for **S.B**).

		Value		Asymptotic Standard Error ^a^		Approximate T^b^		Approximate Significance	
		**S.A**	**S.B**	**S.A**	**S.B**	**S.A**	**S.B**	**S.A**	**S.B**
**Lambda**	*Symmetric*	0.625	0.412	0.138	0.166	3.563	2.067	0.000	0.039
**Goodman & Kruskal tau**	*Scenario (dependent)*	0.380	0.156	0.110	0.093	-	-	0.000 ${}^{c}$	0.008 ${}^{c}$
	*Positioning error (dependent)*	0.637	0.304	0.102	0.127	-	-	0.000 ${}^{c}$	0.009 ${}^{c}$
**Uncertainty Coefficient**	*Symmetric*	0.508	0.216	0.084	0.091	5.597	2.283	0.000 ${}^{d}$	0.003 ${}^{d}$
	*Scenario (dependent)*	0.431	0.184	0.072	0.076	5.597	2.283	0.000 ${}^{d}$	0.003 ${}^{d}$
	*Positioning error (dependent)*	0.618	0.261	0.105	0.114	5.597	2.283	0.000 ${}^{d}$	0.003 ${}^{d}$

*^a^* Not assuming null hypothesis. *^b^* Using the asymptotic standard error assuming the null hypothesis. *^c^* Based on chi-square approximation. ^*d*^ Likelihood ratio chi-square probability.

## Data Availability

Data sharing is not applicable to this article.

## Supplementary Materials

The following are available at https://www.mdpi.com/1424-8220/21/1/273/s1, Figure S1: Calibration of the robot arm and ultrasound (US) probe, Figure S2: Transforming UR5 coordinates to the global reference frame, Figure S3: Design schematic of the ultrasound (US) calibration object, Figure S4: Results of the calibration and acquisition protocol for the Fiber Bragg Grating (FBG)-embedded catheter, Figure S5: The Stewart platform assembly, Table S1: Sources of error and their fractional uncertainties. Accuracies and absolute uncertainties are shown (in mm) for each source. Reconstruction errors are indicated for the stationary (ST) versus Computed Tomography (CT) data, and for the ST versus Visual-Servoing (VS) data, Table S2: Measured pixel distances and pixel density, Table S3: The Stewart Platform Fourier coefficients, and Video S1: Video.mp4.

## Abbreviations

The following abbreviations are used in this manuscript:

CT	Computed Tomography
FBG	Fiber Bragg Grating
PLMs	Periodic Limb Movements
RCCNS	Remote-Controlled Catheter Navigation Systems
US	Ultrasound

## Appendix A. Task-Space Velocity Controller

In (A1)–(A14), all velocities are specified as those necessary to reach the target pose, and their indices (*i*) and those of the error vectors are ignored here for notational simplicity. In Section 3.1, the position error ($E_{p}\in R^{3}$) of the transducer is calculated as

(A1) $$E_{p}=\Delta s_{i}-p_{u},$$

followed by calculating the orientation error ($E_{\theta }={\left[0\;E_{\theta y}\;E_{\theta z}\right]}^{T}\in R^{3}$). Two unit vectors describe the orientation of the transducer: its *z*-axis (${\hat{\theta }}_{z}\in R^{3}$) and *y*-axis (${\hat{\theta }}_{y}\in R^{3}$), respectively (Figure 3a). First, the angular error between the current (*u*) and desired (*i*) *z*-axes is calculated:

(A2) $$E_{\theta z}=\arcsin\left(|\theta_{z}|\right),$$

(A3) $$\theta_{z}={\hat{\theta }}_{u,z}\times {\hat{\theta }}_{i,z}.$$

Likewise, the angular error of the *y*-axes is

(A4) $$E_{\theta y}=\arcsin\left(|\theta_{y}|\right),$$

(A5) $$\theta_{y}=R^{T}\left(E_{\theta z},{\hat{\theta }}_{z}\right)\left(R\left(E_{\theta z},{\hat{\theta }}_{z}\right){\hat{\theta }}_{u,y}\right)\times {\hat{\theta }}_{i,y}).$$

In (A5), we compensate for the error in the *z*-axis through the rotation ($R\left(E_{\theta z},{\hat{\theta }}_{z}\right)\in \mathrm{SO}\left(3\right)$) that rotates ${\hat{\theta }}_{u,z}$ onto ${\hat{\theta }}_{i,z}$, about ${\hat{\theta }}_{z}$. The desired angular velocities ($\omega_{y}\in R^{3}$) and ($\omega_{z}\in R^{3}$) can now be calculated for each axis:

(A6) $$\omega_{y}=\omega_{d}\left(\right|{\hat{\theta }}_{y}\left|\left(1-e^{-E_{\theta_{y}}/\gamma }\right)\right),$$

(A7) $$\omega_{z}=\omega_{d}\left(\right|{\hat{\theta }}_{z}\left|\left(1-e^{-E_{\theta_{z}}/\gamma }\right)\right),$$

which then constitutes the final angular velocity,

(A8) $$\omega_{e}=\omega_{y}+\omega_{z}.$$

In (A6) and (A7), $\omega_{d}$ is the maximum angular velocity of $\theta_{y}$ and $\theta_{z}$ and γ serves as a threshold for the stopping region. To reduce downward surface tension before force control is active, the 2D positioning error is considered, i.e., when (4) becomes $E_{p}={\left[E_{px}\;E_{py}\;0\right]}^{T}$:

(A9) $$V_{e}=V_{d}\left(1-e^{-|E_{p}|/\mu }\right){\hat{E}}_{p}+\left(\tau \int_{t-1}^{t}E_{p}\;dt\right)e^{-\left(\right|E_{p}{\left|/\mu \right)}^{5}}.$$

The constant (μ) is analogous to γ, ${\hat{E}}_{p}=E_{p}/\left|E_{p}\right|$ denotes the direction of the error, $V_{d}$ is the maximum tool velocity, and the term ($1-e^{-|E_{p}|/\mu }$) ensures continuity. The integral time constant (τ) is tuned empirically, and *t* is the updated timestep at each iteration. A discrete setpoint velocity is calculated for force-based explicit force control as

(A10) $$V\left[k\right]=K_{p}E_{f}\left[k\right]+K_{i}V\left[k-1\right],$$

where $K_{p}$ and $K_{i}$ are the proportional and integral gains and *k* describes the discrete-time index. In this case, the positioning error in (A1) changes to $E_{\left|\right|}$, which is the component that is parallel to the surface:

(A11) $$E_{\left|\right|}=E_{p}-\hat{x}\cdot \left(\hat{x}\cdot E_{p}\right),$$

and $\hat{x}$ is the unit vector perpendicular to the surface. The desired velocity is then recalculated by summing (A8) and (A9):

(A12) $$V_{e}=V_{e}+V\left[k\right].$$

The spatial velocity of the robot end-effector comprises (A8) and (A12), resulting in $V_{s}=\left[V_{e}\;\;\omega_{e}\right].$ Utilizing the task velocity in (A9) when the transducer is in the air, and in (A12) for direct contact with the tissue, we prescribe joint velocities ($\dot{q}\in R^{6}$) using the robot Jacobian ($J_{m}\in R^{6\times 6}$) inverse approach, satisfying

(A13) $$\dot{q}=J_{m}^{†}V_{s}.$$

$J_{m}^{†}$ denotes the damped pseudo-inverse of $J_{m}$:

(A14) $$J_{m}^{†}=J_{m}^{T}{\left(J_{m}J_{m}^{T}+\rho^{2}I\right)}^{-1},$$

where ρ is the damping coefficient and $I\in R^{6\times 6}$ is the identity matrix.

## Appendix B. Artificial Arterial Boundary Reconstruction

The model ($D(x_{q})$) from (4) is calculated as

(A15) $$D\left(x_{q}\right)=\frac{f_{q}h_{q}}{\sqrt{h_{q}^{2}\cos^{2}\left(\theta_{q}-\phi_{q}\right)+f_{q}^{2}\sin^{2}\left(\theta_{q}-\phi_{q}\right)}},$$

and relates the states of the ellipse with its radius at the angle $\theta_{q}$. The final estimates of the states (${\hat{x}}_{q|q}$) are:

(A16) $${\hat{x}}_{q|q}={\hat{x}}_{q|q-1}+G_{q}\left(r_{q}-D\left({\hat{x}}_{q|q-1}\right)\right),$$

where $G_{q}$ is the Kalman gain calculated at each iteration (*q*):

(A17) $$G_{q}=P_{q|q-1}J_{q}^{T}S_{q}^{-1}.$$

In (A17), $P_{q|q-1}$ is the covariance of the model prediction, $J_{q}^{T}$ is the Jacobian matrix transpose of $D\left({\hat{x}}_{q|q-1}\right)$, and $S_{q}$ is the covariance of the innovation $\left(r_{q}-D\left({\hat{x}}_{q|q-1}\right)\right)$. Finally, the covariance matrix of the states estimate is updated at each iteration as

(A18) $$P_{q|q}=P_{q|q-1}-G_{q}S_{q}G_{q}^{⊤}.$$

In order to calculate the innovation term (A16), a radius ($r_{q}$) is defined on the first US image (Figure 4b). This radius originates from a 2D seed point obtained from the first known centroid $\left(c_{i=1}=\left[0,c_{y},c_{z}\right]\right)$ and constitutes *M* equi-spaced edge candidates. It has a weighted average length of

(A19) $$r_{q}=\sum_{j=1}^{M}r_{qj}\beta_{qj}.$$

Weights ($\beta_{qj}$) determine the likelihood of a certain candidate (*j*) to be an edge by assuming a normal distribution around the contour of each predicted edge point:

(A20) $$\beta_{qj}=\frac{p_{qj}}{\sum_{j}p_{qj}},$$

where

(A21) $$p_{qj}=\frac{F_{e}{\left(r_{qj},\theta_{q}\right)}^{2}}{\sqrt{2\pi S_{q}}}\exp\left(\frac{-(r_{qj}-D\left({\hat{x}}_{q|q-1}\right)}{2S_{q}}\right)$$

is the probability distribution function of the correct measurement. *F**_e_*(*r**_qj_*, *θ**_q_*) is the edge magnitude at point (*r**_qj_*, *θ**_q_*) and is calculated as an intensity constrained by an upper threshold.
