# Supplementary material for: Real-Time Multi-Modal Sensing and Feedback for Catheterization in Porcine Tissue
Source: Sensors (Basel). 2021 Jan 3;21(1):273. doi: 10.3390/s21010273 (PMC7795440; doi:10.3390/s21010273)
Supplement: Supplementary file 1 [file sensors-21-00273-s001.zip › SUPP/mainsupp.pdf]

Supplementary Materials: Real-Time Multi-Modal Sensing and Feedback for Catheterization in Porcine Tissue

Christoff M. Heunis [1], Filip Šuligoj[1], Carlos Fambuena Santos[1], and Sarthak Misra [1,2]

This material includes relevant explanations, equations, and data files that have been utilized during the main study. These details are regarded as useful for readers aiming to reproduce the calibrations discussed in Section 3 and Section 4. We start off by explaining the data analysis regarding the homogeneous transformations discussed in Section 4. This is followed by calibration procedures, which may be repeated using the ***online data files****. Finally, an overview of the Stewart platform utilized for visual-servo-based motion compensation is provided.*

1. HOMOGENEOUS TRANSFORMATIONS

1.1. Rigid body streaming data

In **Section 4.1**, $\mathbf{R}_i^{\mathcal{P}}$ is defined as the relative rotation matrix of the transformations constructed from the triangulated pose of the precision spheres (as obtained from the NatNet data stream). However, rigid body orientations are only streamed via the Software Development Kit (SDK) toolkit in quaternion format. These quaternions are vectors ($v \in \mathbb{R}^4$) that have to be transformed to their equivalent rotation matrices. The **quaternion** class, specifically, the function *RotationMatrix* provided by the toolkit, is used for obtaining this transformed matrix. With this quaternion and the known position vector from (3), the transformation matrix is calculated using (1) described in **Section III.B**. The SDK is available for Linux, Windows, and Fedora.

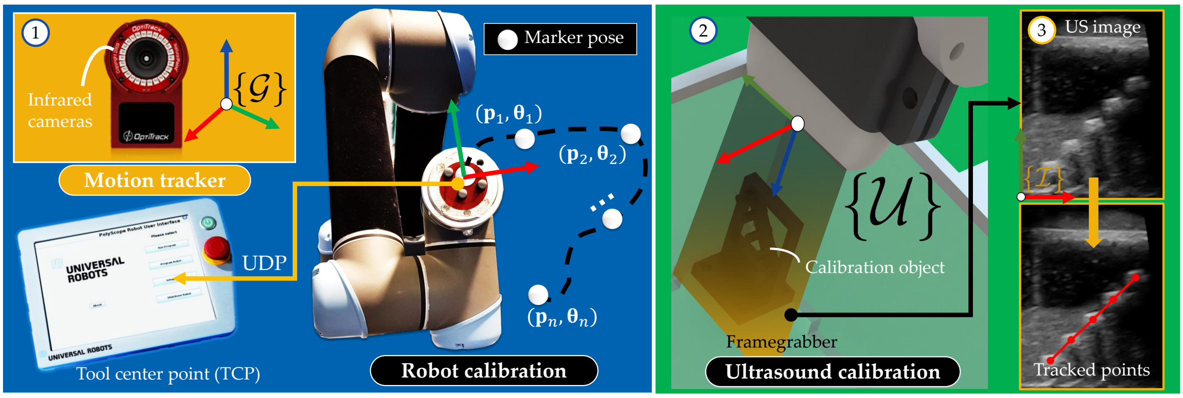

Figure S1. Calibration of the robot arm and ultrasound (US) probe. ① A rigid body precision sphere tool on the manipulator (UR5, Universal Robots, Odense, Denmark) end-effector is tracked using eight infrared cameras (Flex13, Optitrack, Corvallis, USA) that surround the Advanced Robotics for Magnetic Manipulation (ARMM) workspace. The UR5 is sent on a trajectory in 3D space. Each pose containing the end-effector positions (**p**_1, **p**_2, ..., **p**_*n*) and orientations (θ_*u*_1, θ_*u*_2, ..., θ_*u*_*n*) where *n* is the number of samples, are recorded using the infrared cameras. The ground truth for calibration is the pose of the tool center point (TCP) estimated using the low-level controller of the UR5 and streamed via a User Datagram Protocol (UDP) ethernet connection. ② The US probe is calibrated by means of a custom-made calibration object placed inside a gelatin phantom. The US image is recorded via a framegrabber and then processed to map five different points at known coordinates.

1.2. UR5 streaming data

In **Section 4.1**, we consider the end-effector pose of the UR5, expressed as a position ($\mathbf{p}_u \in \mathbb{R}^3$) and angle-axis orientation ($\boldsymbol{\theta}_u \in \mathbb{S}^3$). The latter is an axis-angle rotation unit vector which, similarly to a quaternion vector, can be represented as a 4D vector ($\alpha, [v_x v_y v_z]$) of a 3D rotation. This 4D vector respectively defines the axis ($\mathbf{v} \in \mathbb{R}^3$) of the rotation and the magnitude of the angle ($\alpha \in \mathbb{R}$) at which this vector is rotated. In order to obtain the transformation matrix ($\mathbf{H}_{\mathcal{U}}^{\mathcal{B}}$) by utilizing (1), which describes the configuration of the end-effector w.r.t. the base frame ($\{\mathcal{B}\}$), the equivalent rotation matrix of the transducer ($\mathbf{R}_{\mathcal{U}}$) must be calculated. This is done using Rodrigues formula:

$$\mathbf{R}_{\mathcal{U}} = \boldsymbol{I} + sin(\theta)\boldsymbol{K} + (1 - cos(\theta))\boldsymbol{K}^2 \tag{1}$$

Where $\boldsymbol{I}$ is the identity matrix, θ is the angle of rotation (or modulus of the rotation vector), and $\boldsymbol{K}$ is the skew-symmetric matrix correspondent to the unit vector that points in the same direction as the rotation vector. In this study, this formula is implemented in C++, adapted from the MATLAB® function *rotationVectorToMatrix*.

2. CALIBRATION ERRROR QUANTIFICATION

This section serves to provide a quantitative overview of the sources of error presented in this framework. Reconstruction errors are divided into two groups: those related to the reconstruction of the phantom arteries, and those related to the FBG-embedded catheter. The sources of the phantom reconstruction errors are the UR5 positioning error, the end-effector-to-US probe calibration error, and in-image localization errors. The FBG reconstruction error is the distance between the coordinates in the global reference frame ($\{\mathcal{G}\}$) and their observed coordinates. The fractional uncertainties and sources of error are shown in Table S1.

Table S1. Sources of error and their fractional uncertainties. Accuracies and absolute uncertainties are shown (in mm) for each source. Reconstruction errors are indicated for the stationary (ST) versus Computed Tomography (CT) data, and for the ST versus Visual-Servoing (VS) data.

	Source of error	Accuracy (mm)	Absolute uncertainty (mm)	Fractional uncertainty
Vasculature	ST vs. CT	1.10	0.50	0.45
	ST vs. VS	1.90	0.30	0.16
	Robot Error	0.30	0.10	0.33
Catheter	FBG fiber	0.82	0.21	0.26
	Triangulation	0.56	0.08	0.14

2.1. UR5 base-to-global frame

In **Section 3.2**, to obtain the transformation ($\mathbf{H}_{\mathcal{B}}^{\mathcal{G}}$) from the UR5 base frame ($\{\mathcal{B}\}$) to the global reference frame ($\{\mathcal{G}\}$), we traverse the UR5 end-effector to different poses in the Advanced Robotics for Magnetic Manipulation (ARMM) workspace (Figure S1.1). A rigid body is created in Motive (as explained here) to reconstruct the end-effector trajectory in the global frame. This rigid body provides the triangulated centroid position and rotation between three reflective markers, which are concatenated in a vector that represents the reconstructed pose of the end-effector. The ground truths for the calibration poses are estimated using the low-level controller of the UR5 (**see online URControl.zip**). This controller streams datagrams via a User Datagram Protocol (UDP) ethernet connection to the ARMM computer. Amongst others, these datagrams can be utilized to stream $\mathbf{p}_u$ and

θ_u at a maximum sampling frequency of 125 Hz. After traversing the UR5 end-effector to different poses, $\mathbf{H}_{\mathcal{B}}^{\mathcal{G}}$ is obtained by minimizing the expression

$$\underset{\mathbf{R}_{\mathcal{B}}^{\mathcal{G}},\mathbf{t}_{\mathcal{B}}^{\mathcal{G}}}{\arg\min} \left\| \mathbf{R}_{\mathcal{B}}^{\mathcal{G}} \cdot P_{\mathcal{B}} + \mathbf{t}_{\mathcal{B}}^{\mathcal{G}} - P_{\mathcal{G}} \right\|_2^2 \tag{2}$$

where $\mathbf{R}_{\mathcal{B}}^{\mathcal{G}} \in SE(3)$ is the desired rotation matrix, $P_{\mathcal{B}} \in \mathbb{R}^{3\times n}$ is a matrix containing n samples of end-effector positions in UR5 coordinate system, $\mathbf{t}_{\mathcal{B}}^{\mathcal{G}} \in \mathbb{R}^3$ is the translation vector to be determined, and $P_{\mathcal{G}} \in \mathbb{R}^{3\times n}$ is another matrix containing these positions in the global reference frame. The mean and maximum euclidean errors between the recorded and reconstructed coordinates are 0.32 mm and 0.62 mm, respectively. Figure S2, shows a sample of the recorded end-effector trajectory in both frames, as well as the fitted trajectory.

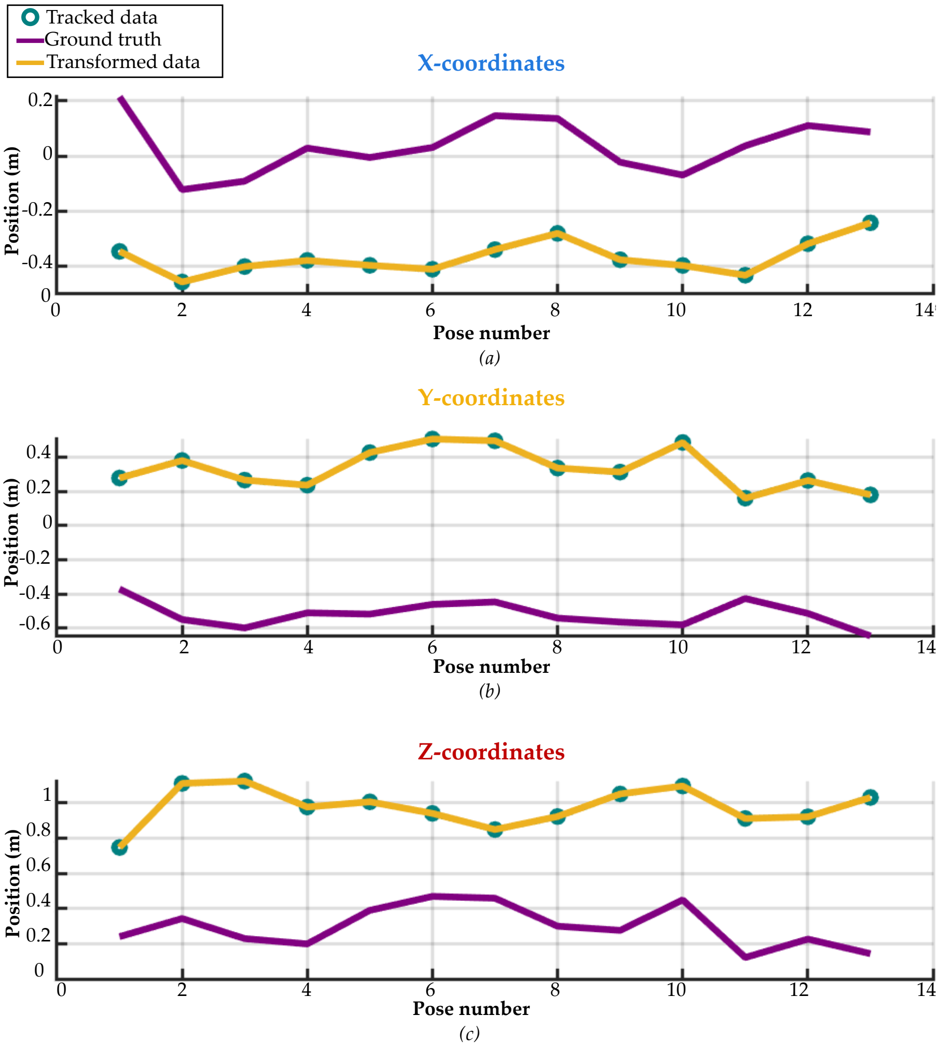

Figure S2. Transforming UR5 coordinates to the global reference frame. *(a)* A sample of X, (b) Y, and (c) Z-coordinates of 13 different end-effector positions in space. The circles represent these positions in the global reference frame as tracked by the cameras, the purple line shows these coordinates in the UR5 base frame, and the yellow line is the fitted data obtained from the calculated transformation matrix.

2.2. Ultrasound polar-to-Cartesian

In Section 4.3, the transformation matrix ($\mathbf{H}_{\mathcal{I}}^{\mathcal{U}}$) requires the pixel density of the acquired US images to be determined. This is done experimentally to localize the polar coordinate US data to Cartesian coordinates. The acquired parameters form part of a function of the acquisition depth, chosen as 90 mm. For this calibration, US acquisition was done inside a phantom that mimics the acoustic characteristics of the tissue used in this study. Computer-aided design (CAD) software (Solidworks, Dassault Systemes, Tennessee) is used to design a calibration object (Figure S1.2), which is 3D printed and covered with gelatin. This design consists of a set of five equispaced cylinders along the diagonal (Figure S3), with known positions and dimensions (**see online calibrator.STL**).

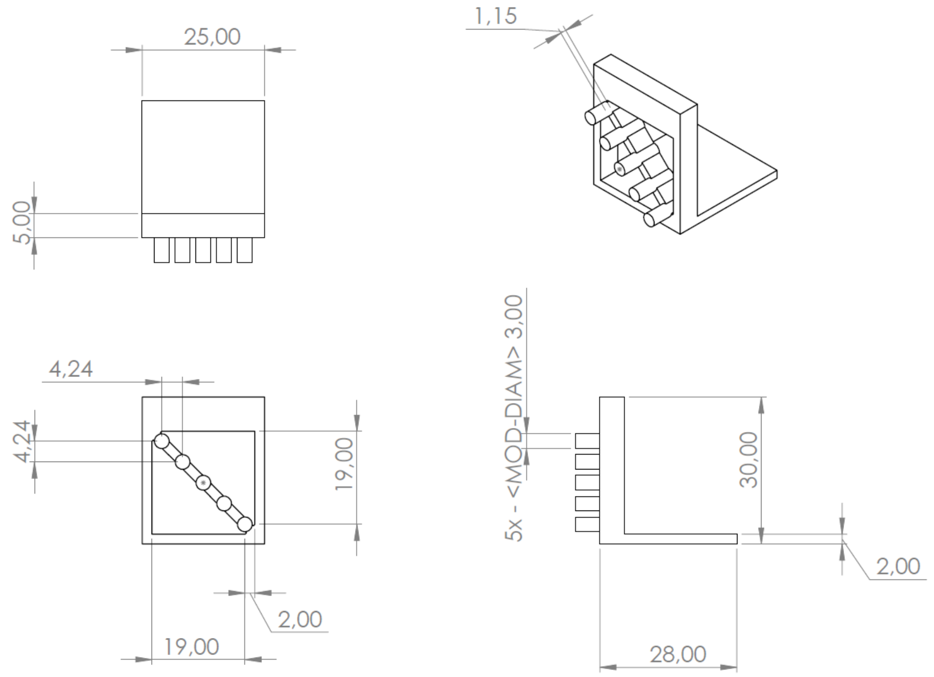

Figure S3. Design schematic of the ultrasound (US) calibration object. Dimensions are shown in mm.

The US transducer is positioned manually above the calibration object and a US image is taken. The acquired image is processed in MATLAB 2019, and each cylindrical cross-section centroid is estimated (**see online calibration.m**). Both the horizontal and vertical distances (in pixels) are averaged to obtain a more robust estimate. The standard deviation of these distances is also computed to estimate the dispersion of the centroids. Table S2 presents the mean and standard deviations of the distances (measured in pixels) and the obtained pixel density for each direction.

Table S2. Measured pixel distances and pixel density. Mean and the standard deviation of the vertical and horizontal distances between cylinders measured in pixels. The real distances in mm and the final pixel density obtained are also shown.

	Mean Dist. (pixels)	Std. Dist (pixels)	Real Dist. (mm)	Pixel Density (pixels/mm)
**X Dir.**	22.30	0.71	4.24	5.26
**Y Dir.**	22.30	0.69	4.24	5.26

2.3. FBG shape-to-base

In **Section 3.2**, we utilize (2) to represent the shape of the Fiber Bragg Grating (FBG)-embedded catheter in the global reference frame. This is preceded by a mold-alignment process as shown in Figure 3. Similarly to (2), a translation and rotation are obtained that minimizes the Euclidean distance between the coordinates of different positions in frames $\{\mathcal{M}\}$ and $\{\mathcal{L}\}$. One hundred points are

interpolated in both the channel and FBG-recorded shapes. Using these points the transformation $\mathbf{H}_{\mathcal{F}}^{\mathcal{C}}$ is calculated by minimizing the following expression:

$$\underset{\mathbf{R}_{\mathcal{F}}^{\mathcal{C}},\mathbf{t}_{\mathcal{F}}^{\mathcal{C}}}{\arg\min} \left\| \mathbf{R}_{\mathcal{F}}^{\mathcal{C}} \cdot P_{\mathcal{F}} + \mathbf{t}_{\mathcal{F}}^{\mathcal{C}} - P_{\mathcal{C}} \right\|_2^2, \tag{3}$$

where $P_{\mathcal{F}} \in \mathbb{R}^{3\times m}$ is a matrix containing m points registered from fiber reconstruction during both insertions (I) and (II), $P_{\mathcal{C}} \in \mathbb{R}^{3\times m}$ is a second matrix of the same size with the points from the fabricated channel shapes, $\mathbf{R}_{\mathcal{F}}^{\mathcal{C}} \in \mathrm{SE}(3)$ is the rotation matrix describing the relative orientation between the denoted frames, and $\mathbf{t}_{\mathcal{F}}^{\mathcal{C}} \in \mathbb{R}^3$ is a vector containing the relative translations. The calibration results of the FBG-embedded catheter are shown in Figure S4. The mean Euclidean error between the actual and reconstructed coordinates is 0.82 mm, with a maximum error of 1.52 mm.

3. VS-BASED MOTION COMPENSATION

In **Section 4.1**, we describe the periodic motions that are introduced to the porcine phantom using a custom-built 6-DoF Stewart platform (Figure S5). The model that describes the periodic motion of the tissue is given by a two-term Fourier series according to

$$\begin{aligned} p[k] = a_{0_x} + a_{1_x}\cos(f_s) + b_{1_x}\sin(f_s) + \\ a_{2_x}\cos(2f_s) + b_{2_x}\sin(2f_s), \end{aligned} \tag{4}$$

$$\begin{aligned} p_z[k] = a_{0_y} + a_{1_y}\cos(f_s) + b_{1_y}\sin(f_s) + \\ a_{2_y}\cos(2f_s) + b_{2_y}\sin(2f_s), \end{aligned} \tag{5}$$

and

$$r_x[k] = A\cos(f_s + c), \tag{6}$$

where $f\_s = \frac{k\omega}{S\_r}$ describes the relation between the discrete time (k), the frequency of the periodic motion per minute ($\omega = \frac{2\pi\omega\_m}{60}$), and the sample rate ($S\_r$). We choose the sample rate to be 100 Hz, and the motions per minute (ω_m) as 20. Furthermore, the amplitude of oscillation (A) is $-5°$ and the phase angle is the starting orientation offset determined by the rigid body orientation explained in Section 1.1.1. The joint angles of the servo motors (defined as $\boldsymbol{\beta} \in \mathbb{R}^6$, where $\boldsymbol{\beta} = [\beta\_1, ..., \beta\_6]$) are calculated using the inverse kinematics of the platform[1], considering the reference signals in (4), (5), and (6). The Fourier coefficients ($a\_0...a\_2$, and $b\_1...b\_2$) are summarized in Table S3.

Table S3. The Stewart Platform Fourier coefficients ($a\_0...a\_2$, and $b\_1...b\_2$) that form the input signals to the motions of the Stewart platform.

	a_0	a_1	a_2	b_1	b_2
p_y[k]	-1.649	0.360	-0.349	-2.613	-0.345
p_z[k]	-3.825	4.079	0.923	-1.266	-0.721

[1] Fichter, E. F. (1986). A Stewart platform-based manipulator: general theory and practical construction. The International Journal of Robotics Research, 5(2), 157-182.

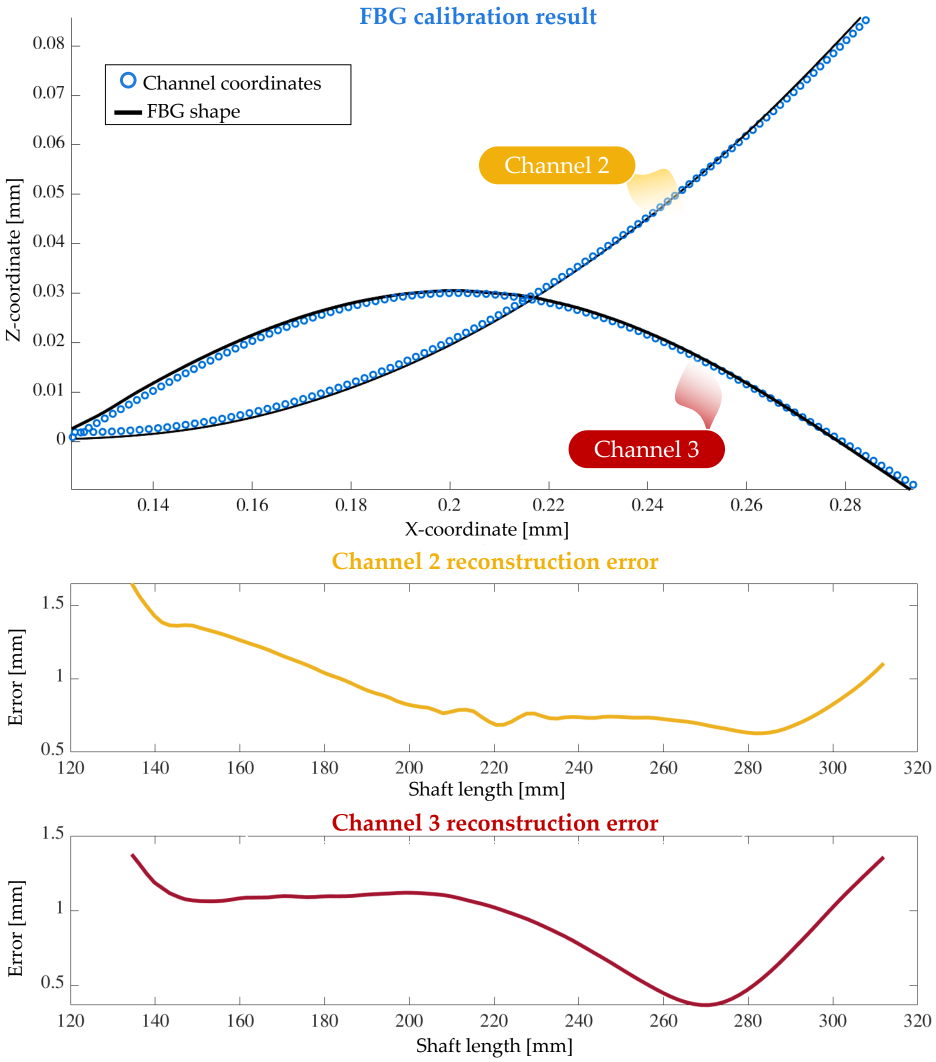

Figure S4. Results of the calibration and acquisition protocol for the Fiber Bragg Grating (FBG)-embedded catheter. Only the section corresponding to the inserted shaft is shown. The errors are indicated in mm for channel 2 (II) and channel 3 (III) of the mold.

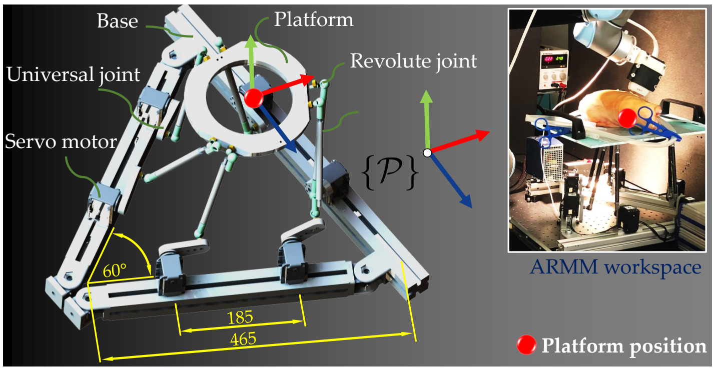

Figure S5. The Stewart platform assembly. The platform dimensions are shown in yellow. It is rigidly mounted on the Advanced Robotics for Magnetic Manipulation (ARMM) workspace tabletop such that the local frame of the platform is aligned with the phantom frame ($\{\mathcal{P}\}$). The center position of the platform is indicated with a red sphere.

Publisher's Note: MDPI stays neutral with regard to jurisdictional claims in published maps and institutional affiliations.
